# The Management of a Pediatric Condylar Fracture With Dynamic Elastic Therapy

**Published:** 2019-09-16

**Authors:** Robert P. Lesko, Brandon J. De Ruiter, George N. Kamel, Edward H. Davidson

**Affiliations:** ^a^Division of Plastic Surgery, Montefiore Medical Center/Albert Einstein College of Medicine, New York, NY; ^b^Department of Plastic Surgery, Case Western Reserve University, Cleveland, Ohio

**Keywords:** mandibular fractures, condylar fractures, pediatric injuries, dynamic elastic therapy, guiding elastics

## CASE DESCRIPTION

**Figure 1:**
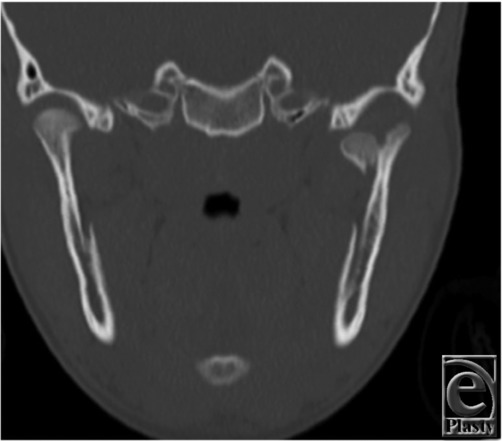
Maxillofacial computed tomographic scan, coronal cut, shows a displaced intracapsular left condylar fracture.

**Figure 2:**
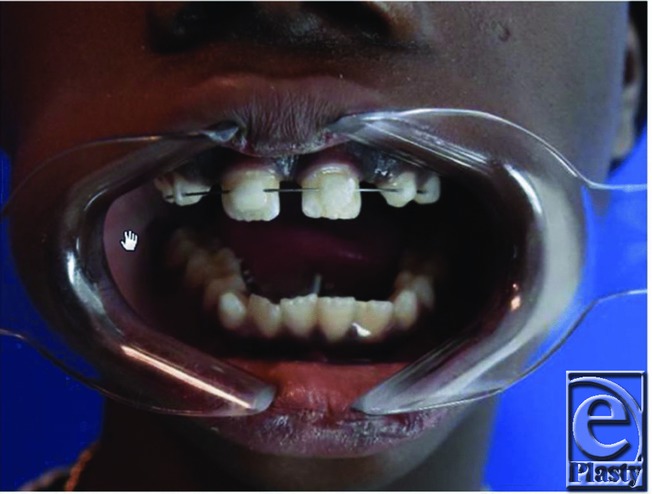
Preoperative intraoral physical examination shows significant malocclusion and chin deviation to the left on mouth opening.

**Figure 3:**
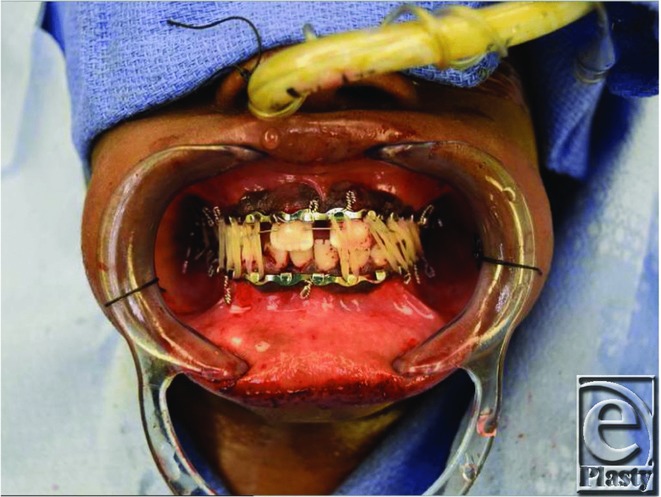
Maxillomandibular fixation with dynamic elastic therapy; *fixating elastics*; class I elastics on the right and class II elastics on the left.

**Figure 4:**
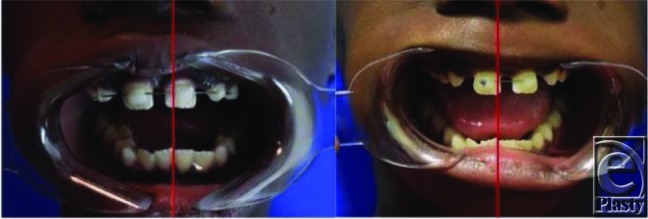
Left image illustrates preoperative malocclusion; right image illustrates midline congruency after completion of dynamic elastic therapy.

An 8-year-old male patient presented after suffering blunt trauma to the left side of the face during a football game. On initial evaluation, the patient denied malocclusion or chin deviation. Computed tomography (CT) identified a displaced intracapsular left-sided mandibular condylar fracture (Fig 1), and conservative treatment was attempted. After 1 week, the patient developed malocclusion and chin deviation (5 mm) (Fig 2) and subsequently was started on a 3-phase protocol utilizing elastic therapy. Phase I used 6-oz ¾-inch “fixating elastics” (class II ipsilateral to injury, class I contralaterally) (figure-of-eight configuration) (Fig 3). In phase II, 6-oz ¼-inch “guiding elastics” were placed (class II ipsilaterally, class I contralaterally) (non–figure-of-eight configuration). In phase III, 6-oz ¼-inch “supportive elastics” were placed (class I bilaterally) (non–figure-of-eight configuration). Each phase lasted 2 weeks, with advancement criteria including centric occlusion without chin deviation. Diet was advanced with phase of therapy from liquid to blenderized to soft. At conclusion of therapy, centric occlusion with congruency of dental and facial midlines (0-mm deviation) was achieved (Fig 4).

## QUESTIONS

1. What is the etiology of pediatric mandibular fractures?
2. How are pediatric patients affected by condylar fractures?
3. What are the different methods of treatment of pediatric condylar fractures?
4. What are the benefits of closed reduction with dynamic elastic therapy?

## DISCUSSION

Several types of traumatic mandibular injury are seen in the pediatric population. According to one recent study, the most common events leading to mandibular fractures were sports injuries, falls, and road traffic accidents.^1^ Anatomic location of pediatric mandibular fractures may vary, but there is an increased incidence of condylar fractures in younger patients and of mandibular angle and body fractures as patients grow older.^2^ Our patient sustained a fracture of the mandibular condyle, the most common type of mandibular fracture in his age demographic.

Several differences exist between pediatric and adult condylar fractures. Pediatric bones are more likely to remodel, which can partially compensate for malunion unlike mandibular fractures in adolescents and adults.^3^ Budding dentition and developing crypts are present in the pediatric mandible, which must be carefully managed or long-term growth issues may occur.^4^ Injuries to the pediatric mandible can have other negative long-term consequences such as facial asymmetry, malocclusion, limited mouth opening, and ankylosis.^5^ These types of injuries may present without subjective malocclusion or chin deviation initially, which highlights the importance of CT as a diagnostic tool. Following trauma, careful clinical evaluation can help prevent these complications.

Treatment modalities for pediatric condylar fractures include conservative management with liquid diet, jaw rest, and careful clinical follow-up, closed reduction with maxillomandibular fixation (MMF), or open reduction with internal fixation (ORIF). Most authors advocate for closed management due to the lack of outcomes data on open surgery and due to the risks of damaging growth centers with open treatment. Remodeling and dental evolution can also overcome malocclusion in closed treatment, especially in patients with primary/deciduous dentition, as there will be more dental evolution compared with patients with secondary teeth.^6^^,^^7^ In one recent study, 50% of pediatric patients with mandibular fracture who underwent ORIF had some type of complication.^6^ After closed reduction, any facial asymmetry may be corrected via orthognathic surgery upon skeletal maturity.

Closed reduction of pediatric condylar fractures with MMF may be accomplished through several methods but can be difficult in pediatric patients when attaching arch bars, brackets, or screws to primary or deciduous dentition. Prior work discusses risks of tooth avulsion and damage to developing dentition.^6^ Traditionally, metal wires have been used to achieve rigid MMF. However, several studies advocate for the use of elastics. One study found no difference in outcomes following dynamic elastic therapy compared with rigid fixation with reduced patient discomfort.^3^ Other benefits of dynamic elastic therapy include customizable management of a healing fracture by altering the vector and degree of traction necessary to restore mandibular vertical height and occlusion while allowing for progressive return of function with less discomfort and decreased risk of ankylosis. This treatment can be extended to patients with bilateral fractures by using class II elastics bilaterally and to more severe cases by substituting class III elastics contralaterally. In pediatric patients, dynamic elastics allow for early jaw mobility and tailored occlusion and are applicable for the management of condylar fractures regardless of the degree of displacement or dislocation.

## SUMMARY

Dynamic elastic therapy is suitable for the management of condylar fractures with any degree of displacement or dislocation in pediatric patients and offers superior or similar results compared with rigid maxillomandibular fixation while minimizing patient discomfort.

## References

[1] Boffano P, Roccia F, Zavattero E (2015). European Maxillofacial Trauma (EURMAT) in children: a multicenter and prospective study. Oral Surg Oral Med Oral Pathol Oral Radiol.

[2] Smartt JM, Low DW, Bartlett SP (2005). The pediatric mandible: II. Management of traumatic injury or fracture. Plast Reconstr Surg.

[3] Noleto JW, Leão EI, Braga CL, Yang S, Sardow A (2011). Conservative approach of condylar fracture in a child by the use of rubber elastics: 7-year follow-up. J Dent Child (Chic, Ill).

[4] Kamboozia AH, Punnia-Moorthy A (1993). The fate of teeth in mandibular fracture lines. Int J Oral Maxillofac Surg.

[5] Wheeler J, Phillips J (2011). Pediatric facial fractures and potential long-term growth disturbances. Craniomaxillofac Trauma Reconstr.

[6] Naran S, Keating J, Natali M (2014). The safe and efficacious use of arch bars in patients during primary and mixed dentition: a challenge to conventional teaching. Plast Reconstr Surg.

[7] Ghasemzadeh A, Mundinger GS, Swanson EW, Utria AF, Dorafshar AH (2015). Treatment of pediatric condylar fractures: a 20-year experience. Plast Reconstr Surg.

